# A Study Protocol for Development and Validation of a Clinical Prediction Model for Frailty (ModulEn): a new European Commitment to Tackling Frailty

**Published:** 2019-01-06

**Authors:** J Apóstolo, E Bobrowicz-Campos, T Moreno-Casbas, L Teixeira-Santos, R Sánchez de Madariaga, E Casado-Ramírez, F Couto, A Coelho, V Parola, I Gil, M Pascual-Carrasco, M de L Almeida, ModulEn consortium

**Affiliations:** 1 Nursing School of Coimbra, Coimbra, Portugal; 2 The Health Sciences Research Unit: Nursing, Nursing School of Coimbra, Coimbra, Portugal; 3 Nursing and Healthcare, Research Unit (Investén-isciii), Madrid, Spain; 4 Frailty and Healthy Ageing-CIBERFES, Madrid, Spain; 5 Telemedicine and e-Health Research Unit (ISCIII), Madrid, Spain

**Keywords:** older adults, frailty, circadian health, predictive model, citizen engagement, digital solutions

## Abstract

There is a growing need to implement and evaluate the technological solutions that allow the early detection of age-related frailty and enable assessment of the predictive values of frailty components. The broad use of these solutions may ensure an efficient and sustainable response of health and social care systems to the challenges related to demographic aging. In this paper, we present the protocol of the ModulEn study that aims to develop and validate a predictive model for frailty. For this purpose, the sample composed by older adults aged 65–80 years and recruited from the community will be invited to use an electronic device ACM Kronowise® 2.0. This device allows proactive and continuous monitoring of circadian health, physical activity, and sleep and eating habits. It will be used during a period of seven to ten days. The participants will also be given the questionnaires evaluating the variables of interest, including frailty level, as well as their experience and satisfaction with the device use. Data provided from these two sources will be combined and the relevant associations will be identified. In our view, the implications of this study’ findings for clinical practice include the possibility to develop and validate tools for timely prevention of frailty progress. In the long term, the ModulEn may contribute to the critical reduction of frailty burden in Europe.

## I. INTRODUCTION

Demographic aging is one of the greatest social and economic challenges of the 21st century. Portugal and Spain are examples of countries in which the phenomenon of aging is particularly worrisome, presenting aging and dependency rates among older adults of 143.9% and 31.4% in Portugal [1] and 120,5 % and 54,1% in Spain [2], respectively. Attending these factors, the prevention of frailty progression has been identified as an emerging priority, due to frailty high prevalence (estimated to be 4 to 17% among community-dwelling older adults aged ≥ 65) [3] and its adverse consequences (falls and fractures, disability, hospitalization, institutionalization and death) [4].

Frailty is a geriatric state defined as a biological syndrome characterized by the decline of various physiological systems due to the accumulation of cellular damage and the inefficacy of repair mechanisms in maintaining homeostasis [5]. There are many external factors that may trigger frailty progression, including socio-demographic and psychological, polypharmacy, comorbidities, and lack of physical activity [6]. Frailty places older people at risk of functional disability in the presence of acute or chronic stressors [7].

The knowledge about factors influencing frailty levels, such as activity/sedentary lifestyle, eating habits and sleep pattern, allows establishing the chronodisruption degree (chronodisruption refers to a prolonged deterioration of the physiological, behavioral and biochemical rhythms of the organism [8]). This knowledge also enables the identification of habits that are susceptible to correction through behavioral changes. Different stimulus and internal/external conditions are able to change the sleep/wake hemostasis. There is also a relationship between changes occurring in the circadian rhythm and activities of daily living that involve excessive light or temperature, leading to a disruption of sleep pattern. These changes may affect significantly the individual’s health and quality of life [9].

The modern developed societies are characterized by a high incidence of chronodisruption due to inadequate exposure to environmental synchronizers. Circadian rhythms are often disturbed by unhealthy behaviors, resulting in a significant mismatch between individual habits (internal time) and the environmental synchronizers (external time), with three coincident trends that impact significantly on society and the health system. The first coincident refers to the more plentiful food (e.g. snack between meals, lack of meal routines). The second coincident describes the hours of sleep that have been progressively reduced along with an increase in irregularity. The third coincident reports the increased exposure to light during the night, with a simultaneous decrease in exposure to bright light during the day. These alterations, frequently present in older people, lead to chronodisruption [6], leading to an increase in the incidence of metabolic syndrome, cardiovascular diseases [10], cognitive impairment [11], affective disorders [12] and some types of cancer [13–16], and playing an important role in aging [17]. The coincident trends alert for the need to consider wearable technologies to track circadian physiology and provide relevant information to be used in health monitoring. These technologies may also be used to get involved people themselves in their healthcare [18].

As the chronodisrruption assessment is still a challenge to be overcome, especially outside the clinical environments, there is a growing need to implement and evaluate new methodologies that can improve objective assessment and early detection of frailty states, in order to prevent or delay its progression [19]. The use of technologies to promote autonomy and well-being in frail persons is seen as a promising methodology, as it fosters definition of solutions adapted to individual needs, monitoring of these solutions implementation and definition of necessary adjustments for optimizing the obtained results. Data on the value of technological solutions integrated into care systems have already been demonstrated and disseminated in a number of reports produced under the European Innovation Partnership on Active and Healthy Aging (EIP on AHA) [20] and the Assisted Ambient Living programs.

The ModulEn pilot project aims to establish a predictive model for frailty as a possible modulator of aging, using data from ACM KRONOWISE® 2.0 sensors. The methodology proposed is non-invasive, has high ecological validity and enables the continuous and proactive monitoring of circadian cycles, sleep patterns, physical activity, eating habits, peripheral body temperature and luminosity to which the sensors users are exposed. The ModulEn project will analyze data obtained from sensors in comparison with retrospective data collected through the questionnaires, promoting the understanding of the frailty-related modulating factors. These data will be used to build and validate a predictive model, and to make it available to the scientific community and health professionals. The transfer of this predictive model into clinical practice will reduce substantially the mobilization of human and material resources, as it will enable the developing of preventive programs, tailored to the older adults’ health needs.

The ModulEn project will collect data on the older adults’ circadian rhythms (recording the peripheral body temperature, motor activity, and body position), levels of exposition to luminosity, chronotypes, sleep and eating patterns and physical activity. Data obtained through sensors will be provided to the study participants in the format of health reports with the personalized counseling on daily life habits. These personalized reports are relevant to increase older adults’ awareness of their unhealthy habits, creating opportunities for establishing pathways for changing their lifestyles.

The ModulEn pilot project is funded by the Fundación General CSIC in the context of the “Interreg España – Portugal” program, and involves several partners from Spain and Portugal, including Nursing and Healthcare Research Unit from the Institute of Health Carlos III as a coordinator, Telemedicine and e-Health Research Unit from the Institute of Health Carlos III, Chronobiology Laboratory of the University of Murcia and the Nursing School of Coimbra. The information presented in this paper refers to clinical twin studies carried out in Spain and Portugal.

## II. METHODOLOGY

### Sample

This multicenter observational descriptive study will include community-dwelling participants, aged 65–80 years, recruited by family nurses from primary health centers in territories of Huelva, Ponferrada, and Lugo in Spain, and through cultural and sports associations, municipal services, health- and day centers in the Central Region of Portugal. The main criteria for inclusion will be the absence of moderate to severe cognitive decline and/or the absence of the unstable clinical condition. All participants will be voluntaries and will give their written consent to be a part of this study.

### Sample size and power analysis

Considering an alpha level of 0.05 and a confidence interval of +/−0.04 in bilateral contrast for a proportion (without prior estimation), and assuming a maximal drop-out rate of 6%, a global sample of 640 subjects will be employed to reach at least a number of 601 participants.

### Data collection

After providing sociodemographic, anthropometric and clinical data, eligible participants will provide information on their circadian state, physical capacity, sleep and eating habits, and frailty. Then, they will be invited to use ACM Kronowise® 2.0 sensors. Finally, the participants will be asked to report any adverse events that may occur during the device utilization, and share their experience with the device use. Study data will be collected and managed using a secure, web-based application Research Electronic Data Capture (REDCap) tools hosted at the Institute of Health Carlos III [21].

### Instruments

Data collected in Portugal and Spain will refer to the same variables. However, attending cultural differences, it was necessary to include some country-specific variables.

#### 1. Assessment tools used in both participating countries

**Circadian system and sleep** will be assessed based on the following instruments:

- the Epworth Sleepiness Scale (ESS), a self-administrated questionnaire measuring daytime sleepiness [22] and the likelihood of falling asleep in particular situations;
- the Pittsburgh Sleep Quality Index (PSQI) assessing sleep quality and disturbance over the last month [23];
- the reduced version of the Morningness-Eveningness Questionnaire (MEQ) estimating the individual preferences towards morning-type, evening-type, or intermediate-type in circadian rhythms based on self-description [24], adapted for use in the populations of older adults by the Laboratorio de Cronobiología de la Universidade de Murcia.

**Eating Habits** will be assessed through a questionnaire on types and times of meals taken on workdays and days off.

**Frailty**. For the assessment of frailty, the criteria from the phenotypic model proposed by Fried [25] will be used. The participants will be given the FRAIL scale consisting of five direct questions assessing fatigability, resistance, ambulation, illness, and loss of weight [26]. The FRAIL score ranges from 0 to 5, with 0 indicating robust health status, 1 and 2 indicating pre-frailty and 3, 4 and 5 indicating frailty.

**Physical activity** will be assessed using a short version of the International Physical Activity Questionnaire (IPAQ) [27], developed for older adults (www.ipaq.ki.se). This instrument collects data on activities of different intensities carried out in the last seven days and about their frequency and duration.

**ACM Kronowise® 2.0**, an electronic non-invasive device assessing biological rhythms (for more details see https://www.um.es/cronobiologia/en/what-we-do/applied-research/), will be used to collect data on the functioning of the circadian system through a series of sensors that record peripheral temperature, body position, physical activity, environmental light exposure, and sleep patterns. The recording of data will last seven days.

**End-user experience** with the use of the device ACM Kronowise® 2.0 will be collected through a questionnaire developed for the purpose of this study. The questionnaire consists of open-ended questions that identify the positive and negative aspects of device use, and that address the individual’s readiness to integrate digital health-related tools into daily life.

#### 2. Country-specific procedures

**Screening procedures** will be implemented exclusively in the study conducted in Portugal. To evaluate the eligibility of older adults, the 6-item Cognitive Impairment Test (6CIT) [28], validated for Portugal [29] will be administrated. The screening assessment will also include two open-ended questions that examine the overall state of health and explore the level of autonomy in the instrumental activities of daily living.

**Frailty w**ill be additionally assessed through a questionnaire on fatigue and objective measures regarding weight loss, walking speed and handgrip. For physical activity assessment, the IPAQ-SF (as mentioned above) will be used. The detailed information on the Portuguese version of the assessment procedures with respective cut-off points for each of the five symptoms is provided elsewhere [30].

### Data analysis

Data obtained through questionnaires will be analyzed using the IBM SPSS Statistics 24.0. For a summary presentation of the characteristics of the study participants, a descriptive analysis of the variables will be performed. The qualitative variables will be examined based on the Chi-square test or Fisher’s exact test. The corresponding tests will be used to compare quantitative variables in the subgroups of the study sample. A p value ≤ 0.05 will be considered statistically significant. For the analysis of the association between variables of interest adjusted for covariates, multivariate logistic regression models will be built. Data obtained through electronic devices will be analyzed using the Circadianware software, based on the time series analysis. Finally, predictive modeling using data mining techniques for weighted integration of all parameters of interest will be applied. The parameters considered for this model will include circadian rhythms, intake times and physical activities.

### Model development

In order to implement the clinical predictive model for frailty, four supervised ML algorithms will be used. These algorithms will be based on the techniques of Naïve Bayes [31], Decision Table [32], J48 Decision Trees [33] and Multilayer Perceptron [34]. The information collected will be used for the construction of two data sets: (i) one matrix including data on circadian rhythms and physical activity [35]; and (ii) a frailty vector including the state of robustness-frailty for each individual.

In order to evaluate and compare the four ML algorithms, 10-fold cross validation methodology [36] will be implemented. Each ML algorithm will be performed using the global matrix and frailty vector data, divided into “training” subset and “test” subset. In the first phase, the system will be trained based on the data from the “training” subset. Then, the “test” subset will be used as input to the trained system. The second phase will result in a definition of a hypothetic frailty vector for the individuals considered in the “test” subset. This vector will be compared with the real frailty vector obtained from the same subset. The process will be repeated 10 times using ten different random “train” and “test” subsets. The precision and recall of the statistical measures [36] resulting from each of the 10 trials will be averaged in order to compute the effectiveness of each ML algorithm. Then, a single global measure (F-measure), including precision and recall, will be computed for each ML algorithm. The algorithm with the highest F-measure will be selected as the best algorithm to predict frailty.

### Ethical considerations

The implementation, evaluation, and documentation of the study will be carried out in accordance with the Declaration of Helsinki. In order to ensure the confidentiality and privacy of the data obtained, security measures for coding, storage, distribution, and protection will be taken to prevent unauthorized access to databases, according to with the General Data Protection Regulation.

The study was approved by the Ethics Committee of the Health Sciences Research Unit: Nursing of Nursing School of Coimbra (Opinion no. 510/06-2018), and the Ethics Committee of the Institute of Health Carlos III (CEI PI 56_2018-v3). This endorsement was valid for the Nursing and Healthcare, Research Unit and the Telemedicine and e-Health Research Unit, both of them dependent on the Institute of Health Carlos III. Moreover, all Spanish Primary Health Centres that have agreed to participate as recruiters have the approval of their corresponding Local Ethics Committee, and all Portuguese institutions that have decided to collaborate in the study gave the formal authorization for its realization.

## III. DISCUSSION

ModulEn pilot project will develop and validate a new clinical prediction model to inform decision-making in healthcare. The diffusion of this model among health and social care professionals may facilitate the definition of integrated and personalized approaches to frailty that, in turn, will allow the maximization of disability-free life expectancy. The maximization of older person’s functional ability and preservation of their independence and autonomy for as long as possible are considered as priority goals by different international entities, including World Health Organization (WHO) and EIP on AHA [37,38]. These entities argue that older people need to be supported to benefit from the opportunities offered by increased life expectancy. The advantages resulting from this support include prevention of decline in physical and cognitive ability, maintenance of an active and meaningful life, and improvement of quality of life of the older person and his/her family. In a larger perspective, the maximization of a disability-free life expectancy may contribute to the maintenance or improvement of the efficiency and sustainability of health and social care systems that, nowadays, are threatened by the challenges of demographic aging [39]. However, the integration of the recommendations defined by WHO and EIP on AHA into a daily practice of health and social care professionals may be extremely difficult without the parallel change of health paradigm from acute intervention to prevention [40]. For this purpose, there is a need to emphasize a holistic approach to health, with the active involvement of health and social care providers, fostering healthy, active, participatory and inclusive aging in place. In the last years, several international projects proposing a community-led, preventive, personalized and participatory approach to aging were implemented [41]. Some of them have indicated technological solutions as crucial to comprehensive geriatric care.

In this context the research on frailty seems to be crucial as it enables shaping of a new model for screening, treatment, and monitoring of this clinical condition [42] and as it sensitizes stakeholders for a necessity to include societal, community and clinical contexts to respond to the person’s real needs and to increase the transparency and impact of the treatment proposed [43]. The recent findings on frailty have shown that the new model of integrated care should prioritize the encouragement and empowerment of older people and their families to take responsibility for their own autonomy and well-being [43]. These encouragement and empowerment actions may include raising public awareness about successful aging, including behavioral change, and promotion of healthy lifestyles. However, to make this happen, consistent evidence on the association between lifestyle factors and frailty needs to be generated and transferred to the clinical practice. The present study will provide this evidence, contributing to the delineation of new approaches to successful aging.
